# Nurturing others neglecting self: A qualitative study on work-life balance among nurse educators at a Gauteng nursing education institution

**DOI:** 10.4102/curationis.v49i1.2816

**Published:** 2026-03-11

**Authors:** Letta Mathebula, Rirhandzu F. Mathevula, Tshiamo N. Ramalepa

**Affiliations:** 1 Department of Nursing Science, School of Health Care Sciences, Sefako Makgatho Health Sciences University, Pretoria, South Africa

**Keywords:** work-life balance, nurse educators, academics, factors, nursing educational institution, scoping review

## Abstract

**Background:**

Nurse educators are instrumental in preparing the next generation of nurses. However, the changing landscape of nursing education is making it difficult for them to exemplify and advocate for work-life balance (WLB). Nursing education institutions face challenges with limited resources and increasing student numbers, making educators crucial for maintaining high educational standards. Despite this, there has been limited research on how educators manage the overlapping pressures of their careers and personal lives.

**Objectives:**

This study explored the experiences of nurse educators regarding WLB at a nursing education institution in Gauteng province, South Africa.

**Method:**

A qualitative, exploratory, descriptive and contextual design was employed. Purposive sampling was used to select 16 nurse educators across four campuses. Data collection involved face-to-face individual semi-structured interviews, and data analysis followed a qualitative thematic approach.

**Results:**

This research revealed two themes: Heavy workload and responsibilities, and Blurred boundaries between work and personal life. The spillover of work demands into personal time, particularly those driven by student expectations, emerged as a significant challenge to achieving a balanced work-life dynamic.

**Conclusion:**

Addressing the challenges faced by nurse educators in achieving WLB requires more than individual resilience. There is a need for comprehensive support systems and strategic interventions to help nurse educators manage their responsibilities effectively and achieve a healthier balance between work and life.

**Contribution:**

This study could assist larger efforts to create supportive and sustainable work environments for nurse educators, which would benefit the educators and further the development of the nursing profession.

## Introduction

Work-life balance (WLB) is a critical issue for nurse educators globally because of limited resources, increasing student enrolment, and growing professional expectations that exacerbate challenges. The quality of work life among nurses is shaped by a complex array of factors, including personal characteristics, occupational demands and work environment, and psychological aspects that impact mental well-being and job satisfaction (Sibuea, Sulastiana & Fitriana 2024). Rohini, Elankoon and Kisokanth (2024) investigated the intersection of quality of life and WLB among Sri Lankan nurse educators, noting a moderate degree of reciprocity between their work and personal domains. This phenomenon highlights the significance of attaining a balance between professional obligations and personal life, a critical factor in determining job satisfaction and overall well-being within the nursing profession. Notably, studies focusing on nursing students have identified a significant relationship between WLB, academic stress and the impact of supervisory support on their overall well-being (Mudzi, Jiyane & Sepeng 2022). Nurses often neglect their own well-being while prioritising patient care and other work-related nursing activities, a trend worsened by crises such as the coronavirus disease 2019 (COVID-19) pandemic. The nursing philosophy of care, emphasising compassion and Ubuntu, can paradoxically hinder nurses’ self-care, making it challenging for them to prioritise their own needs. This pattern is evident in high-pressure settings, such as nursing education institutions and clinical environments (Thaba-Nkadimene et al. 2021). Nurse educators may blame themselves for WLB issues, rather than seeing those issues as symptoms of larger systemic problems. Moreover, they are expected to balance roles as role models, mentors and academic leaders while managing teaching, research and clinical duties.

This complex role often leads to significant stress and a lack of institutional support, resulting in persistent workload pressure, emotional fatigue and a diminished separation between work and personal life (Erasmus, Downing & Ntshingila 2024). A recent scoping review by Mathebula, Mathevula and Ramalepa (2025) highlights occupational stress, understaffing and poor boundary-setting as key factors contributing to work-life imbalance among nurse educators in sub-Saharan Africa. Notably, it also identifies a significant research gap in understanding specific challenges faced by nurse educators in South Africa, calling for context-specific studies to develop effective interventions (Mathebula et al. 2025). Nurse educators play a crucial role in shaping the future of nursing by training the next generation of nurses (Jobst et al. 2022). Traditional gender roles further complicate the situation, as the majority of nurse educators are women who often bear additional domestic and caregiving responsibilities (Matahela & Van Rensburg 2021). Poku et al. (2023) emphasised that nurse educators struggle to achieve WLB without institutional support such as paid parental leave and flexible work arrangements. However, they face significant challenges that impact their well-being and job satisfaction, particularly in South Africa (South African Nursing Council [SANC] 2020). The nursing sector in the country struggles with severe staff shortages, high nurse-to-patient ratios and limited career advancement opportunities (Mthimunye & Daniels 2020). These systemic issues place immense pressure on nurse educators, who must train students under difficult conditions while managing their heavy workloads (September & Steyn 2022).

The challenges faced by nurse educators are complex and multifaceted (Boamah et al. 2024). This includes excessive workload because of staffing shortages, inadequate institutional support and emotional stress from demanding academic and clinical responsibilities (Boamah et al. 2024). Moreover, poor WLB among nurse educators may lead to detrimental effects, such as increased stress, emotional exhaustion, decreased life satisfaction and compromised physical health. Heavy workloads and the blurring of boundaries between work and personal life significantly contribute to these problems, elevating the risk of burnout (Pasay-ana et al. 2020). A study by Matahela (2025) found that accreditation requirements, changing industry standards, and the need to keep curricula current add to workload and stress levels upon nurse educators. The global nursing shortage has worsened these challenges, placing even greater demands on nurse educators (Morris 2023). As the need for qualified nurses increases, nurse educators must train more students with fewer resources (Jobst et al. 2022). Nurse educators report the feeling of being undervalued and overwhelmed, with little time left for professional development or personal well-being (Erasmus et al. 2024). Therefore, addressing challenges requires more than individual coping mechanisms; instead, organisational changes such as better policies and supportive leadership are needed to create a healthier work environment. On the other hand, supportive measures such as paid parental leave, affordable childcare and mental health resources can help alleviate some of the pressures they face (Boamah et al. 2024; Rinne et al. 2024).

According to Morris (2023), compassionate leadership recognises the contributions of nurse educators to teaching and learning, and the reduction of bureaucratic burdens is also essential. Additionally, providing opportunities for professional development and workload management training can empower nurse educators to thrive in their roles (Jobst et al. 2022). Meanwhile, Erasmus et al. (2024) highlight the need for institutions to foster job satisfaction, reduce burnout and promote WLB among nurse educators by providing a supportive environment, ultimately aiding in their long-term retention. However, many institutions fall short of offering sufficient support, forcing nurse educators to manage these challenges without assistance. El-Awaisi et al. (2020) and Rawlinson et al. (2021) found inadequate interprofessional support to be creating significant challenges for nurse educators, including role ambiguity, professional identity issues and lack of recognition. This may lead to collaboration barriers, increased stress and workload, ultimately compromising WLB and overall well-being. Hierarchical structures and insufficient institutional support mechanisms exacerbate these issues.

The absence of adequate support for nurse educators negatively affects not only their own well-being but also compromises the quality of nursing education and, by extension, patient care (SANC 2020). South Africa’s national strategies emphasise the need to reform nursing education by integrating innovative pedagogical models, digital technologies and fostering a conducive work environment. This paradigmatic shift is anticipated to promote the well-being of nurse educators, thereby enhancing the overall quality of nursing education provision (Cambaza 2023; Department of Health 2020; Lovern et al. 2021). It is important to critically examine challenges to formulate strategies that enhance the WLB of nurse educators (Poku et al. 2023). Therefore, this study explored the experiences of nurse educators regarding WLB at a Nursing Education Institution in Gauteng province, South Africa. The outcomes of this research might contribute to broader initiatives aimed at establishing sustainable and supportive work environments for nurse educators, thereby benefiting both the educators and the ongoing advancement of the nursing profession.

## Research methods and design

### Research design

A qualitative, exploratory, descriptive and contextual design was utilised. This research design guided the researchers in planning and implementing the study using a technique suitable to explore the experiences of nurse educators regarding the WLB. The study was conducted at a specific nursing education institution (NEI), a government-run organisation with six campuses that collaborates with different universities and provides nursing qualifications that align with the National Qualifications Framework (NQF). It operates within the Gauteng Department of Health (GDoH) and is subject to the regulatory oversight of the SANC and the Council on Higher Education (CHE) for quality assurance purposes.

### Population and sampling

The population for this study consisted of nurse educators. The target population consisted of nurse educators with 6 months or more of experience working at a designated nursing education institution in Gauteng province. The accessible population was further defined as nurse educators with teaching experience who were directly involved in student education at the selected institution and were present on the day of data collection. A non-probability purposive sampling method was employed to choose nurse educators who seemed knowledgeable and experienced about WLB. The sample size for the study was 16 nurse educators. The sample size was determined by data saturation, ensuring that the collected data were comprehensive and rich enough to address the research objectives. Saturation occurred when no new information emerged. The researcher observed saturation during the 13th interview and then conducted three more interviews to confirm data saturation.

### Data collection

The data collection method for this study involved conducting face-to-face, semi-structured individual interviews. The researcher utilised a semi-structured interview guide with both open-ended and closed-ended questions to obtain demographic data. The demographics included age, gender, qualification and years of experience as a nurse educator. The interview guide was piloted before data collection to ensure it elicited rich responses and provided clear guidance. During this phase, leading questions, such as ‘Do you have a work-life balance policy in your institution?’ were identified and revised to open-ended forms like ‘Tell me about your work-life balance policy in your institution’. The first three interviews were included in the study, as the guide required only minor adjustments and met qualitative standards. This process refined the guide to better capture participants’ lived experiences. A central question was posed to the participants, and probing techniques were used to gather more in-depth information from the participants during the interviews. The central question read as follows: ‘What are your experiences as a nurse educator regarding work-life balance?’

Participants were provided with an information letter that outlined details about the study, and thereafter signed a consent form. The interviews took place in quiet, isolated environments provided by managers of different campuses to minimise interruptions. All interviews were conducted in English, the formal language of communication used by the nurse educators who worked in the selected NEI. Interviews were recorded with the participants’ permission, and field notes were also taken. Each interview session lasted for a duration of 30 min – 45 min, allowing participants to provide detailed responses.

### Data analysis

A qualitative thematic approach, following six steps, was employed in the data analysis. Thematic analysis was conducted through a multistep process: data immersion via transcript readings, coding of significant features, theme identification and refinement, and theme definition and naming (Naeem et al. 2023). The researchers provided both the audio-recorded interviews and transcripts to an independent coder for additional analysis. This coder, working independently from the research team, ensured greater credibility of the coding process and the overall findings. After completing the initial coding, the coder shared the results with the researchers, and they then engaged in a collaborative review. During this process, they reflected on coding strategies, addressed challenges and discussed key insights. Detailed discussions were held on selected portions of the data, allowing both parties to compare coding decisions and underlying justifications. Any differences in interpretation were resolved through open and reflective dialogue, strengthening methodological rigour and deepening understanding. This collaborative approach ultimately resulted in consensus on necessary refinements and the identification of four central themes, each supported by related sub-themes.

### Ethical considerations

Ethical clearance to conduct this study was obtained from the Sefako Makgatho University Research Ethics Committee on 19 September 2025. The ethics approval number is SMUREC/ H/249/2023:PG.

## Results

The demographic information presented reflects that the majority of participants were female, most of whom were in their 50s when it comes to age. Their educational qualifications ranged from bachelor’s degrees to doctoral levels, reflecting varied academic backgrounds. Participants’ professional experience spanned from 1 year to 21 years, representing both early-career and seasoned nurse educators. The sample also included one male participant.

Two themes emerged from data obtained from the 16 participants. The themes are further supported by sub-themes. The two themes are: Heavy workload and responsibilities, and blurred boundaries between work and personal life. In Theme 1, heavy workload and responsibilities, nurse educators reported feeling overwhelmed by the demands of their roles, which hindered their personal growth and were made worse by the various responsibilities they balance in both their work and social lives. Theme 2, blurred boundaries between work and personal life, highlighted the problem of workload spillover; nurse educators found it difficult to separate their professional duties from their time because of the demands from their students. These challenges indicate that nurse educators struggled to manage their workloads while maintaining a healthy WLB.

### Theme 1: Heavy workload and responsibilities

Theme 1 revealed that the participants felt exhausted because of their workload and numerous roles within the NEI. This made it difficult for them to balance work and personal life. As a result, they had little time for family, professional development and personal pursuits. This led to emotional depletion, stagnation and neglect of their overall well-being. Consequently, their physical and mental health, relationships and job satisfaction suffered. A vicious cycle of burnout and exhaustion ensued. Theme 1 is supported by three sub-themes: feeling overwhelmed with workload demands, workload interference with personal life and managing multiple work and life roles.

#### Sub-theme 1.1: Feeling overwhelmed with workload demands

Participants revealed that excessive workloads and responsibilities left them feeling overwhelmed, making it challenging to manage their workdays effectively, meet deadlines and maintain WLB. They mentioned difficulties in planning their workdays, having to take work home to meet deadlines, struggling to submit work on time, and feeling like WLB is non-existent because of constant demands and a lack of autonomy in managing their workload. The following verbatim quotes provide the voices of the participants:

‘You can’t always plan your workday effectively, and with deadlines for marking tests and workbooks, you often end up taking work home to catch up.’ (Participant 2, female, 55 years, Baccalaureus Curationis in Nursing Education [B. Cur N. Ed])

‘Another challenge is submitting work on time due to being overwhelmed with work and other responsibilities. You sometimes fail to meet deadlines.’ (Participant 4, female, 54 years, B. Cur N. Ed)

‘As a nurse educator, I feel like work-life balance doesn’t exist. The current situation is all about work, with constant demands and new programmes adding to our load, leaving us overwhelmed and without a say in managing our responsibilities.’ (Participant 11, female, 55 years, B. Cur N. Ed)

In contrast to the widespread experience of overwhelming demands, one participant reported a surprisingly manageable workload, achieving a sustained WLB throughout their extended period of service:

‘I had to take work home, but it was not excessive. It would only take about 1.5 hours to complete. After nine years at this X campus, I have never felt overwhelmed. My work-life balance is good, and I can easily complete small tasks at home to stay ahead.’ (Participant 12, female, 63 years, B. Cur N. Ed)

#### Sub-theme 1.2: Workload interference with personal life

Participants reported that their WLB was complicated by the need for personal development, as pursuing further studies and staying updated in their field added to their workload. They emphasised the importance of continuous learning but struggled to find time for professional development outside of working hours. The following verbatim quotes represent the voices of the participants:

‘It’s not balanceable, on top of it I’m studying, that it’s, it’s not easy, umm at work what are used to do I used to take a lot of work wanting to be superman [*mmm*] it’s almost seeing what other lectures are going through and now taking on their work [*mm*], and can’t keep the peace to try keep the moral.’ (Participant 1, female, 36 years, Master’s degree in Nursing Education [M. N. Ed])

‘You need to be a step ahead of your client who’s the student, so that means you’ve got to keep developing, keep reading and that keep reading in inverted commas also has to do with that work life balance, because now, when would you say you are now reading, You can’t do it in your 8 to 4 role because there are so many activities that are involved in our work.’ (Participant 7, female, 56 years, Doctor of Philosophy [PhD])

‘I’m struggling with personal development because I’m expected to advance in my work and pursue further studies, all while managing family responsibilities. I am caught between three competing demands: work, studies, and family. To balance these, I must be available for all three, ensuring that work does not suffer while I prioritise studying and spending time with my family.’ (Participant 16, male, 53 years, M. N. Ed)

#### Sub-theme 1.3: Managing multiple work and life roles

The findings from the sub-theme managing multiple work and life roles revealed that participants struggled to balance multiple roles, feeling overwhelmed by the demands of work, family and personal development. They reported challenges in managing responsibilities as professionals, family members and individuals, leading to conflicts and difficulties in maintaining quality and managing workload effectively. The participants alluded to saying the following:

‘You feel overwhelmed, it is challenging to juggle between work and social activities, and you may fail to complete your tasks at home as a mother and wife.’ (Participant 5, female, 55 years, PhD)

‘My experience as a lecturer has shown that you leave work tired because you do not have a break. The current curriculum structure is overwhelming and congested. We are expected to juggle teaching, simulations, clinical supervision, assessment, and feedback without adequate breaks or support, making it challenging to maintain quality and manage our workload effectively.’ (Participant 6, female, 54 years, M. N. Ed)

‘I have a daughter and an elderly mother who relies solely on me. I need to make time for them. I’m also actively involved in my church. With only Saturday and Sunday off, it means juggling all these roles.’ (Participant 7, female, 56 years, PhD)

### Theme 2: Blurred boundaries between work and social life

Participants experienced blurred boundaries between work and social life, leading to WLB challenges. Administrative demands and after-hours student contacts intruded on their time, causing role interference and straining relationships. The overwhelming workload compromised their ability to manage responsibilities at home, resulting in difficulties balancing work and social life. The participants mentioned the workload spillover, and students demand confirmation of this. Theme 2 is supported by two sub-themes: workload spillover and increased students’ demands and expectations.

#### Sub-theme 2.1: Workload spillover

The sub-theme ‘workload spillover’ revealed that participants experienced work demands spilling over into their personal lives, causing them to struggle in fulfilling their family roles. Furthermore, the participants expressed that excessive workload led to working late hours, which affected their ability to care for their families. They also mentioned that their family responsibilities, such as caring for children, spouses and elderly parents, suffered as a result. The following statements from the participants supported the workload spillover:

‘Yes, When I’m at home I had to cater for the needs of my family my three kids, my husband and being also to cater for my father who is old, so that is another role that I need to fulfil but sometimes it can get so much whereby the demands at work overlap and I cannot give my fullest to my, to my family, yah and in that case they suffer.’ (Participant 1, female, 36 years, M. N. Ed)

‘I think the three hours given to us were to assist us to balance work and social life … prepare lessons, but now, because of more work allocated to us, we even work late at night and are unable to take care of children and family.’ (Participant 3, female, 47 years, B. Cur N. Ed)

‘Yes, I have to carry work, mark and lesson preparations, you also need to be a mother, grandmother at home, and not a lecturer.’ (Participant 10, female, 63 years, B. Cur N. Ed)

‘I can say another challenge is that you find that you didn’t get enough time to prepare, as you receive the PowerPoint late and you don’t get enough time to dwell into them before you go to class.’ (Participant 13, female, 54 years, M. N. Ed)

‘I am going to do every day before I start my day from 07h00 until 16h00 so that I don’t take my work home, but there are instances where you are forced to take your work home, and that is how my family life gets affected. There are instances where you do not have a choice but to take the work home.’ (Participant 15, female, 43 years, M. N. Ed)

#### Sub-theme 2.2: Increased students’ demands and expectations

The sub-theme ‘increased students’ demands and expectations’ reveals that participants experienced pressure to be constantly available to students, often beyond working hours, which further blurred the lines between work and personal life. This demand to respond to students’ needs and queries impacted their family life, causing feelings of neglect and mistrust among family members, and added to their workload stress. The following quotes represent the voices of the participants:

‘The more the work spills into family life, the more my family suffers, feeling neglected and unimportant. They blame work for household issues, accusing me of prioritising it over them, and losing trust, wondering if there are more important things or people in my life.’ (Participant 16, male, 53 years, M. N. Ed)

‘So the other thing that could maybe although I cannot call it the challenge, because now students most of the time are calling after hours so in trying to make sure that they understand maybe a certain portion of the content, but I would do that with pleasure, because now I understand that yes, maybe he or she is busy reading then she can’t or he can’t understand, so I will gladly explain whatever the student is asking.’ (Participant 8, female, 57 years, Bachelor of Arts with Honours degree)

‘If there are no structures in place and you are not well orientated, you feel empty in you because you won’t be able to answer students when they ask questions, and if they can see that you are clueless, they will lose trust in you.’ (Participant 10, female, 63 years, B. Cur N. Ed)

‘We must do the preparations, because even here at work, we do not have time to do other things when you are still marking, the students come in. You leave your preparations, and then you concentrate on that.’ (Participant 11, female, 55 years, B. Cur N. Ed)

## Discussion

The purpose of the study was to explore the experiences of nurse educators regarding WLB at a nursing education institution in Gauteng province. The findings of the study revealed that the multifaceted responsibilities of nurse educators within NEIs present significant challenges that affect their work performance and social lives. These challenges are deeply intertwined with the demands of teaching, mentoring, administrative tasks and institutional expectations. McCarthy and Fitzpatrick (2019) highlight that the diversity and intensity of these duties often hinder nurse educators from achieving a WLB. The accumulation of professional obligations frequently leads to extended work hours, which, in turn, limit opportunities for social interactions and family engagements (Erasmus et al. 2024). This eventually leads to stress because workload-related stress is a recurring theme in the nursing education environment. Rensburg and Kolade (2023) emphasise how the high demands placed on nurse educators blur the lines between work and social life, adversely affecting their emotional health and job satisfaction. Without effective time management strategies or established boundaries, educators are vulnerable to burnout (Mathebula et al., 2025; Taylor & Forrester 2024). Institutional and societal expectations surrounding student engagement and academic outcomes add to another factor of pressure. Kim and Asbury (2019) argue that heightened expectations diminish nurse educators’ professional fulfilment, often resulting in emotional fatigue. Similarly, Savolainen and Malinen (2019) call for comprehensive institutional support, particularly in curriculum development, to alleviate psychological distress and enhance operational efficiency. Despite these demands, the implementation of support remains inconsistent, contributing to the stress faced by nurse educators.

Pennbrant and Svensson (2018) advocate for the inclusion of autonomy, flexible work conditions and access to professional development as key factors that can improve nurse educators’ experiences. Moreover, Brauner et al. (2022) further highlight the value of strong administrative and peer support, coupled with effective time management practices, in promoting productivity and reducing stress. Compounding these issues is the persistent faculty shortage within nursing education. Triyawan (2022) and Owens (2017) argue that the convergence of teaching, research and administrative responsibilities exacerbates job-related stress and impairs job satisfaction. The COVID-19 pandemic intensified these pressures, exposing the undervalued status of nurse educators and reinforcing the urgent need for empathetic, responsive leadership (Kim & Asbury 2019). Yet, as Savolainen and Malinen (2019) suggest, many institutions have yet to implement effective, long-term strategies to address these challenges.

Administrative dynamics and structural limitations play a critical role in shaping nurse educators’ capacity to manage work and social responsibilities. Pennbrant and Svensson (2018) underline the importance of supportive administrative relationships, realistic workload expectations and firm boundary setting to enhance nurse educators’ ability to navigate work demands. To address these complex and evolving challenges, Ramachandaran, Pillay and Govender (2024) propose the implementation of institution-specific strategies tailored to the unique needs of nurse educators. Such targeted strategies are essential for creating supportive and sustainable academic environments, ultimately promoting job satisfaction, engagement and WLB. Blurred boundaries between work and social life have emerged as a significant challenge to achieving WLB among nurse educators. Studies consistently report that spillovers from work responsibilities, particularly persistent student demands and after-hours communication, play a central role in this challenge. Nurse educators often take unfinished academic tasks home to meet deadlines while simultaneously managing student inquiries outside regular working hours. This aligns with the negative spillover theory, which posits that when professional responsibilities encroach upon personal time, the distinction between work and social life erodes (Li, Chen & Zhao 2024).

To address these challenges, Thomson, Jacobs and Mthembu (2022) underscore the importance of setting and maintaining clear boundaries to mitigate negative spillovers. Their research advocates institutional-level interventions, including formal policies designed to discourage communication after business hours (Thomson et al. 2022). Such policies reinforce professional boundaries and promote sustainable workload practices. Building on this, Hurd and Singh (2021) argue for a transformative shift in academic culture, suggesting that critical reflexive practice can help higher education institutions challenge long-standing norms that perpetuate harmful work expectations. This approach may foster a culture rooted in mutual support and equity, ultimately creating more sustainable environments for educators. Time management and the ability to assert personal boundaries are also recognised as crucial strategies for supporting WLB. Weberg, Mangold and Harvey (2021) emphasise that structured scheduling and thoughtful prioritisation of tasks enable nurse educators to effectively manage the diverse and often conflicting demands of their roles. However, poor time management and excessive multitasking may escalate conflict between professional obligations and personal responsibilities, undermining composure and performance in both domains. Chan et al. (2019) reinforces this, noting that the absence of clear demarcations between work and home life directly increases stress levels. They advocate for intentional efforts by nurse educators to define boundaries more rigorously, safeguarding both professional effectiveness and overall well-being.

Workload spillovers pose significant challenges to achieving a WLB, as demanding jobs can encroach on personal time and strain emotional well-being (Paquette, Trépanier & Ménard 2025). This can lead to emotional exhaustion, lower relationship quality and diminished social support (Reimann et al. 2022). Excessive work commitments can also reduce meaningful family interactions. Greater workplace autonomy and supportive policies, such as flexible schedules and remote work options, can enhance employee well-being and promote healthier family dynamics (Mohite & James 2024; Zychová, Fejfarová & Jindrová 2024). Achieving WLB is a growing concern, as work-family conflict continues to blur the boundaries between professional responsibilities and home life. Disruptions in this balance can strain both parenting effectiveness and job performance, often resulting in burnout and diminished personal satisfaction (Wickramasinghe & Nakandala 2022). For working-class individuals in particular, the emotional toll may include elevated levels of anxiety and depression (Mohite & James 2024). Encouragingly, career adaptability and the individual’s ability to adjust and respond to changing work demands have shown promise in mediating these challenges, offering a potential pathway to regain equilibrium and improve employee outcomes (Nurcholidah et al. 2023).

The shift to remote work brought on by the COVID-19 pandemic has exacerbated these challenges. The integration of work into domestic settings has significantly extended the workday, diminishing boundaries between professional and personal domains. Moorhouse, Wong and Ho (2022) revealed that approximately 74% of nurse educators admitted to checking work-related emails after hours, a practice indicative of prolonged work engagement and the erosion of traditional workday boundaries. This pervasive connectivity contributes to ‘tele pressure’, the compulsive urge to respond promptly to work communications, even during personal time (Setyaningrum 2023). The consequences of telepressure are particularly acute among female faculty, who comprise a substantial 89% of the nurse educator workforce, as gendered expectations and caregiving responsibilities often compound professional commitments (American Association of Colleges of Nursing 2022). Yedidia, Bickel and Schwartz (2021) emphasise that women, particularly within academia, report heightened difficulty in disengaging from professional responsibilities while at home, especially without a dedicated workspace or structured work hours. Moorhouse et al. (2022) reinforce that the dissolution of physical barriers between professional and personal environments intensifies WLB conflicts. These findings underscore the need for institutional recognition of remote work’s impact on nurse educators, especially regarding gendered experiences and the psychological toll of continuous availability. Strategies to mitigate the pressure and promote work-life separation, such as setting email curfews, encouraging asynchronous communication, and promoting boundary-setting, are essential for the long-term well-being and sustainability of the academic nursing workforce.

### Limitations

The study’s findings are contextual and should be interpreted with caution because of several limitations. Data collection was restricted to two campuses within a single College of Nursing in Gauteng, South Africa, because of logistical and administrative constraints. The qualitative design and limited sample scope render the results non-generalisable. Additionally, reliance on self-reported data obtained through interviews may have introduced recall bias, further contextualising the findings.

## Conclusion

The study purpose was achieved by exploring the experiences of nurse educators regarding WLB at a nursing education institution in Gauteng province. The findings of the study suggest that nurse educators face many challenges in achieving WLB. Heavy workloads, blurred boundaries between work and social life, and multiple responsibilities make it difficult to manage both professional and personal demands. These issues can lead to stress and lower job satisfaction. Support from NEIs is important. Time management and setting clear boundaries can help reduce the negative effects of work-life imbalance. When nurse educators have these supports, they are better able to balance their roles. By understanding these challenges and offering practical solutions, NEIs can help nurse educators achieve a WLB.
